# To What Affections of the Lungs Does Bronchitis Give Origin?

**Published:** 1861-09

**Authors:** 


XII.—*To what Affections of the Lungs does Bronchitis give origin?* Prize Essay. By DANIEL DENISON SLADE, M.D., of Boston. 8vo.

DR. SLADE had no competitors for the prize of one hundred dollars, offered by the Massachusetts Medical Society for the best dissertation on the subject referred to in the title of this notice. We will not say that *therefore* the committee unanimously adjudged him that amount, for his essay is a carefully-prepared paper; but in the total absence of competition, it was not likely that much difference of opinion could exist among the members of the committee. There is this point to remark, however, that, being the only essay presented, it must have been thoroughly scrutinized by each of the Prize Committee, and therefore the compliment paid to Dr. Slade is the more marked.

A paper written to order, and not springing spontaneously from the writer's own suggestive mind, ought not to be expected to be eminently original. Authorities have to be ransacked to see if the subject for a prize, which may never have occupied more than a moment of the writer's consideration previously, has not been worn threadbare already, or to discover if new paths can be opened out by new researches. We may, therefore, expect to find frequently abstracts of the opinions of others rather than purely original papers. This remark only partially applies to the essay before us; for it would be unjust to the author were we to pass over the evidences which are presented of a practically inquisitive mind and an extended appreciation of the importance of pathological phenomena.

The principal subjects investigated are: collapse of the lung as consequent upon obstruction of the bronchi; its causes; the effects of inspiration and expiration in their power to get rid of bronchial obstructions; the mechanical condition, conducing to the production of collapse, to be found in the air-tubes themselves, etc. The writer arrives at the following conclusions:—

That the production of collapse of the lung is due, first, to the existence of mucus in the bronchi, which is the more liable to produce collapse in proportion as it is tenacious; secondly, to weakness or inefficiency of the inspiratory power, however it may be caused; thirdly, to inability to cough or to expectorate, and thus remove the obstructing mucus.

The pathology of bronchial abscess as related to bronchial collapse; the conflicting views of authors on the question, "Does bronchitis give rise to true pneumonia, lobular as well as lobar?"; the secondary and more permanent lesions of the lung, the result, for the most part, of bronchitic collapse, and leading to atrophy of the lung, and vesicular emphysema as a secondary effect of bronchitis, are also discussed at some length. The writer thinks that vesicular emphysema *may* be produced by the expiratory act, but that, in addition to either this or the inspiratory act, another condition is necessary to the perfection of the theory, and this is to be found in the partially diminished bulk, or, in other words, pulmonary collapse or permanent atrophy of some portion of the lung.

The effects, on the lung, of contraction, obliteration, and dilatation of the bronchi are alluded to; the relation of asthma to bronchitis, and of the latter to phthisis. Humoral asthma is undoubtedly owing, in many cases, to a spasmodic derangement of the muscular fibres of the bronchi, whereby a great accumulation of mucus takes place, since in health the removal of this mucus depends upon the regular action of these fibres. Bronchitis is neither a direct nor a frequent cause of phthisis; and is to be so considered only when a predisposition exists.

